# Whole microbial community viability is not quantitatively reflected by propidium monoazide sequencing approach

**DOI:** 10.1186/s40168-020-00961-3

**Published:** 2021-01-21

**Authors:** Ya Wang, Yan Yan, Kelsey N. Thompson, Sena Bae, Emma K. Accorsi, Yancong Zhang, Jiaxian Shen, Hera Vlamakis, Erica M. Hartmann, Curtis Huttenhower

**Affiliations:** 1 Department of Biostatistics, Harvard T.H. Chan School of Public Health, Harvard University, 655 Huntington Avenue, Boston, MA 02115 USA; 2 Broad Institute of MIT and Harvard, 415 Main Street, Cambridge, MA 02142 USA; 3 Department of Civil and Environmental Engineering, Northwestern University, 2145 Sheridan Road, Evanston, IL 60208 USA; 4 Department of Immunology and Infectious Diseases, Harvard TH Chan School of Public Health, Harvard University, 655 Huntington Avenue, Boston, MA 02115 USA

**Keywords:** Propidium monoazide, 16S rRNA-sequencing, Built environment communities

## Abstract

**Background:**

High-throughput sequencing provides a powerful window into the structural and functional profiling of microbial communities, but it is unable to characterize only the viable portion of microbial communities at scale. There is as yet not one best solution to this problem. Previous studies have established viability assessments using propidium monoazide (PMA) treatment coupled with downstream molecular profiling (e.g., qPCR or sequencing). While these studies have met with moderate success, most of them focused on the resulting “viable” communities without systematic evaluations of the technique. Here, we present our work to rigorously benchmark “PMA-seq” (PMA treatment followed by 16S rRNA gene amplicon sequencing) for viability assessment in synthetic and realistic microbial communities.

**Results:**

PMA-seq was able to successfully reconstruct simple synthetic communities comprising viable/heat-killed *Escherichia coli* and *Streptococcus sanguinis*. However, in realistically complex communities (computer screens, computer mice, soil, and human saliva) with *E. coli* spike-in controls, PMA-seq did not accurately quantify viability (even relative to variability in amplicon sequencing), with its performance largely affected by community properties such as initial biomass, sample types, and compositional diversity. We then applied this technique to environmental swabs from the Boston subway system. Several taxa differed significantly after PMA treatment, while not all microorganisms responded consistently. To elucidate the “PMA-responsive” microbes, we compared our results with previous PMA-based studies and found that PMA responsiveness varied widely when microbes were sourced from different ecosystems but were reproducible within similar environments across studies.

**Conclusions:**

This study provides a comprehensive evaluation of PMA-seq exploring its quantitative potential in synthetic and complex microbial communities, where the technique was effective for semi-quantitative purposes in simple synthetic communities but provided only qualitative assessments in realistically complex community samples.

Video abstract

**Supplementary Information:**

The online version contains supplementary material available at 10.1186/s40168-020-00961-3.

## Introduction

While culture-independent methods provide a diverse toolbox for characterizing microbial communities, it remains challenging to differentiate viable or metabolically active microbes from nonviable community members [1]. The functions and phenotypes of microbial communities are arguably defined by biochemically active (“viable”) microbes, but assays such as high-throughput sequencing typically assess “dead” microbes as well [2]. This difference can be critical in environments such as the built environment (BE), where desiccation, nutrient scarcity, unusual chemical profiles, frequent disinfection practices, and lack of autochthonous microbiota can contribute to much greater prevalence and abundance of dead microbes than in more natural settings where microbial communities typically evolved [3, 4]. This, in turn, has phenotypic consequences in settings where dead microbial measurements can bias or overwhelm assessments of viable community members [5]; or in contexts such as hospitals or clean rooms, where viable microbes are of much greater importance than nonviable ones [6]; or in BEs where antimicrobial resistance, genetic mobility, or pathogens only pose a human health risk if viable [5, 7]. While many methods can determine the viability of individual microbes—including, obviously, culture—very few are available, and none have been well-validated, for determining whole-community viability profiles [1].

One method used for community viability characterization is high-throughput sequencing combined with a chemical treatment to deplete signal from extracellular or nonviable (“relic”) DNA [2, 6, 8]. The most common representative is propidium monoazide (PMA) treatment, a DNA-intercalating dye that is membrane-excluded by viable cells but can be photoactivated to bind unprotected DNA molecules [6]. This method has been used in combination with molecular readouts such as polymerase chain reaction (PCR), amplicon (16S rRNA gene) sequencing, or shotgun metagenomics to selectively detect viable (membrane-intact) microbes without signals from non-viable (membrane-compromised) ones in various environmental communities, to determine the relationship between viable microbial diversity and environmental conditions [2, 6, 9, 10]. When used for whole-community assessment, PMA treatment is typically followed by 16S rRNA gene amplicon sequencing (here “PMA-seq”), which (among other examples) has been used to determine the viable community first in water samples [11] and cleanroom environments [6]; applied to human stool to evaluate fecal microbiota transplantation protocols [10, 12]; and to sputum specimens from cystic fibrosis patients to elucidate the viable community related to the disease [9].

While PMA-seq has been used in a variety of contexts, the accuracy of such measurements has not been well evaluated. In particular, it is unknown which and what fraction of dead cells from different microbes are removed by the treatment, or whether the viable fraction is correspondingly unaffected. PMA treatment was originally developed to be used with universal or targeted PCR readouts [13, 14], and its combination with sequencing has been relatively limited. Notably, amplicon sequencing itself is often non- or semi-quantitative, which is only sometimes acknowledged during its use. Extraction bias, amplification, 16S rRNA gene copy numbers, bioinformatics classification accuracy, and of course (non)viability all influence the degree to which it truly quantifies a community’s taxonomic profile [15]. Additionally, PMA treatment, though easy to perform, lacks a standardized procedure for its application in different sample types or under different conditions, a known confounder [6, 16]. Dye concentrations ranged from 25 to 150 μM in previous studies; light intensity, incubation time, and temperature are all variable; and different microbes, at different abundances, in different surrounding biochemical matrices, can be differentially affected [5, 6, 10, 16]. While available evaluations explored its potential as a qualitative method [17], it is not at all clear whether PMA-seq reliably quantifies viable microbial communities (to the degree that this is possible via sequencing-based assays).

In this study, we thus provide the first systematic evaluation of PMA-seq as a semi-quantitative approach to characterize viability in complex microbial communities. We first observed the effects of PMA-treatment in a simple co-culture of *Escherichia coli* and *Streptococcus sanguinis*. The treatment was then applied to complex communities spiked with known controls, and a range of transit environmental communities, followed by 16S rRNA gene (“16S”) sequencing for taxonomic profiling. The results were compared with several previous PMA-seq studies of clean room dusts, human stool, and soil samples [2, 5, 10]. This ultimately showed PMA-seq to be reliable in low-complexity communities, but largely inconsistent in realistic microbial communities, although interestingly these inconsistencies were themselves relatively similar across studies.

## Results

### PMA treatment successfully depletes signal from relic DNA in simple synthetic communities

**Fig. 1:**
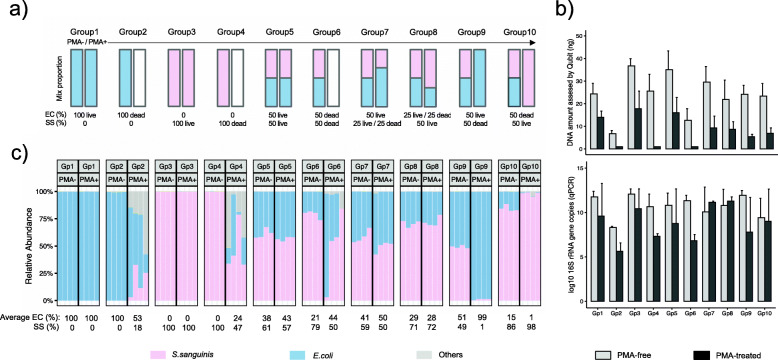
PMA-seq identified viable microbes in simple synthetic communities. **a** Expected community structures of our ten live/dead *Escherichia coli* and *Streptococcus sanguinis* mixtures before and after PMA treatment: group (1) 100% live *E. coli;* 100% *E. coli* after PMA treatment; (2) 100% dead *E. coli*; 0% *E. coli* after PMA treatment; (3) 100% live *S. sanguinis*; 100% *S. sanguinis* after PMA treatment; (4) 100% dead *S. sanguinis*; 0% *S. sanguinis* after PMA treatment; (5) 50% live *E. coli* and 50% live *S. sanguinis*; same proportion after PMA treatment; (6) 50% dead *E. coli* and 50% dead *S. sanguinis;* no nucleotides after PMA-treatment; (7) 50% live *E. coli*, 25% live *S. sanguinis* and 25% dead *S. sanguinis*; 67% *E. coli* and 33% *S. sanguinis* after PMA treatment; (8) 25% live *E. coli* and 25% dead *E. coli*; 50% live *S. sanguinis*; 67% *S. sanguinis* and 33% *E. coli* after PMA treatment; (9) 50% live *E. coli*, 50% dead *S. sanguinis*; 100% *E. coli* after PMA treatment; 10) 50% dead *E. coli* and 50% live *S. sanguinis*; 100% *S. sanguinis* after PMA treatment. **b** DNA quantity (ng) (top) and 16S rRNA gene copy numbers (bottom) of the 10 synthetic cultures with and without PMA treatment. Error bars represented the standard deviations. **c** Relative abundances of synthetic community members by PMA-seq before and after PMA treatment. Each experiment was carried out in quadruplex

Our evaluation of PMA’s ability to deplete signal from relic DNA started with the validation in simple synthetic communities, where we built cultures with known mixtures of living and heat-killed *Escherichia coli* and *Streptococcus sanguinis* (Fig. 1, “Methods”). The two species were chosen as representatives of Gram-negative and Gram-positive strains, respectively, known to be a major determinant of PMA susceptibility [13, 17, 18]. In communities composed of mostly living cells (Fig. 1a, b, groups 1, 3, and 5), PMA treatment resulted in lowered DNA yields, suggesting the existence of non-viable or damaged cells in these cultures or the non-specific effects of PMA treatment to viable cells (though in these communities 16S gene copies did not vary significantly; paired *t* test, *p* = 0.36, 0.27, and 0.46 for groups 1, 3, and 5, respectively). DNA yields and 16S gene copy numbers both decreased after PMA treatment in the heat-killed synthetic communities (groups 2, 4, and 6), as expected, though the killing process itself likely induced some DNA degradation.

Sequencing results (Fig. 1c) suggested that signals from relic DNA were removed in cultures with only one viable microorganism (groups 9 and 10). In monocultures containing only one non-viable microorganism (groups 2 and 4), which would ideally result in "no" sequenced DNA, the small number of sequences still obtained were (as expected) from either the non-viable bacteria added or from the one that was not added, likely due to low-level contamination or bleed-through from other samples (as would occur in any near-empty amplicon library, regardless of viability). Most importantly in such a setting, the overall depletion of relic DNA in these groups (2 and 4) was supported by much lower DNA yields and qPCR signals. PMA treatment of mixed communities comprising two microorganisms (groups 7 and 8) was qualitatively successful but different from the target mix proportions. Particularly, we expected a ~ 30% reduction in DNA yield and 16S gene copies after PMA treatment, while we did observe less DNA after PMA treatment (Fig. 1b, top), the resulting 16S copies (Fig. 1b, bottom) and the ratio of the two species (Fig. 1c) were not consistent. It is worth noting that this inconsistency may not lay in PMA treatment alone, as any amplicon-based taxonomic profiling tends to be affected by factors such as PCR efficiency during target amplification and library preparation, 16S rRNA gene copies in different taxa, and the overall composition of microbial community [19]. Taken together, though, these results indicated that PMA-seq was able to successfully reconstruct the communities of viable/heat-killed *E. coli* and *S. sanguinis* in the ten synthetic communities, regardless of Gram stain, with some degree of quantitative accuracy (Fig. 1).

### PMA-seq does not accurately quantify microbial viability in spiked complex communities

**Fig. 2:**
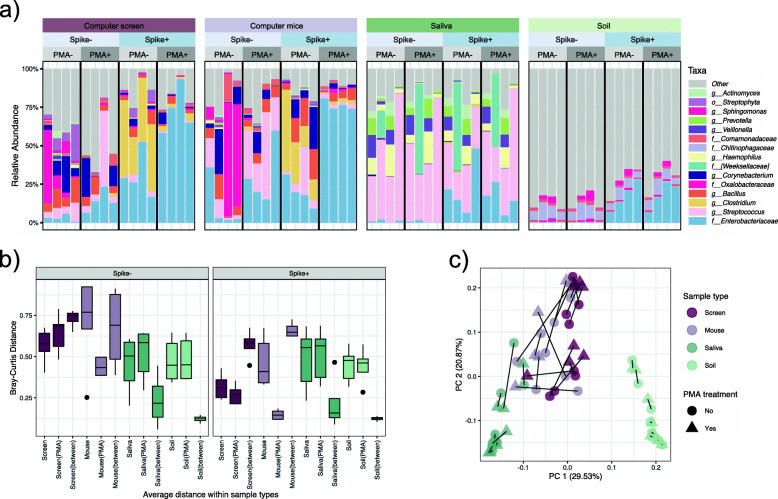
PMA-treatment resulted in different degrees of compositional changes in different complex communities. Our second evaluation of PMA-seq used four environmental microbial community types (high and low biomass, high and low expected viability) spiked with varying levels of cultured / heat-killed *E. coli*. **a** Relative abundances of 15 taxa detected with the highest mean abundance across all samples. Each sample type contains four biological replicates. **b** Bray-Curtis distances within and between community samples with PMA treatment and samples without PMA treatment. Columns labeled with the sample type alone (e.g., screen) show dissimilarities within the indicated PMA-free samples. Those annotated “type(PMA)” (e.g., screen(PMA)) show calculations within the PMA-treated samples, and “type(between)” (e.g., screen(between) represents distances between paired samples with and without PMA treatment. **c** After constructing an ordination based on each sample pairwise Bray-Curtis dissimilarity, variation across the first explanatory axes is largely separated by human-associated uses, while the second axes appear to be explained by sample biomass. Here, lines connect identical samples with and without PMA treatment

To further test the accuracy of PMA-seq, we evaluated its performance by spiking control microbes into complex microbiomes from a variety of environments (Fig. 2). Two were high-biomass, high-complexity communities (i.e., soils and human saliva), and two were representatives of low-biomass communities sourced from expected low-viability environments (i.e., computer screens and computer mice) (“Methods,” Additional file 1: Figure S2). Additionally, we spiked each of these samples with different concentrations of a 1 ml mixture of live/dead *E. coli* cells at the ratio of 1:1 (“Methods”) to assess whether PMA treatment was able to effectively remove known relic DNA within a complex community.

Within these community types, PMA-seq was unable to denote microbial viability quantitatively, with its performance largely dictated by each environmental source’s characteristics, which added to existing distortions from amplicon-based sequencing (Fig. 2). The low biomass samples indicated larger compositional dissimilarities between PMA-free vs. PMA-treated samples. Conversely, only minor changes were observed in the spiked and resident microbes of the high biomass samples (Fig. 2b). Sample type, as expected, accounted for most of the observed differences in these communities, explaining 46.5% of the variation (PERMANOVA FDR adjusted *p* = 0.0015) (Additional file 4). The effects of PMA-treatment differed significantly by sample types (PERMANOVA FDR adjusted *p* = 0.039), arguably more effective in low biomass samples, in which somewhat fewer competing microbial, chemical, and matrix-driven effects were present to prevent intercalation and nucleotide depletion.

To explore the potential of PMA-seq as a semi-quantitative tool, we compared the abundance of known spike-in cultures with and without PMA treatment. The addition of *E. coli* controls to spiked-in (“Spike+”) groups boosted the abundance of Enterobacteriaceae, as expected. Indoor samples (computer screens and computer mice) were further enriched for *Clostridium* species in the PMA-free groups, possibly resulting from contamination in these samples (Fig. 2a). Further increases of Enterobacteriaceae were observed upon PMA treatment in the Spike+ groups, likely resulting from the elimination of a subset of dead microorganisms from the community, as expected.

It should be noted that the abundance change of a taxon in PMA-seq only represents a “relative viability,” where an increased abundance could be due to the taxon being “more viable” or from the changes of other microbes. We thus calculated normalized PMA efficacies independently in the four sample types based on the relative abundance of Enterobacteriaceae, combined with the 16S rRNA gene copies obtained from qPCR in the four aliquots of samples (“Methods,” Additional file 3). Briefly, to determine the amount of correct PMA tagging (efficacy) in each sample, we divided the amount of dead Enterobacteriaceae that was successfully removed after PMA treatment by the total amount of spiked Enterobacteriaceae. The efficacy should equal to 0.5 under ideal conditions, given that each spike-in culture was a mixture of live/dead *E. coli* cells at the ratio of 1:1; an efficacy over 0.5 indicated the unintentional removal of viable microbes, while below 0.5 suggests incomplete depletion of non-viable cells. The high efficacy in computer screens (1.01), computer mice (0.96), and soil (0.87) samples suggests a partial toxicity of PMA to viable cells, which somewhat explained the drastic compositional changes in the communities from screens and mice. The efficacy in saliva samples is relatively low (0.35), indicating the incomplete elimination of relic DNA. These results reiterated that PMA-seq did not accurately quantify microbial viability in complex communities, with efficacy varying in different sample types.

### Complex microbial communities from the BE do not respond consistently to PMA

**Fig. 3:**
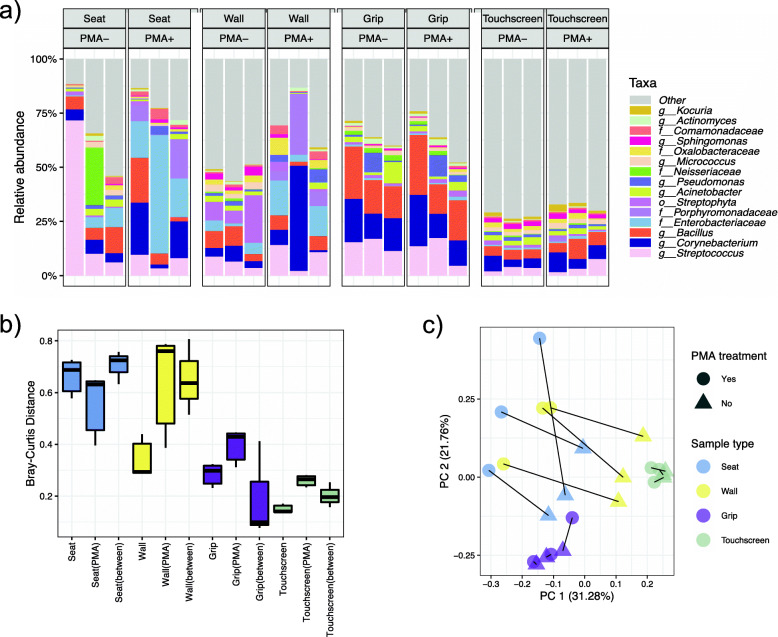
Effects of PMA treatment on microbial communities from the Boston MBTA transit environment. **a** Relative abundances of 15 taxa with the highest means across four BE sample types with and without PMA treatment. Each column represents a biological replicate. **b** Bray-Curtis dissimilarity distributions between MBTA samples with and without PMA treatment. Columns labeled with the sample type alone (e.g., seat) show distances within that PMA-free group; “type(PMA)” (e.g., seat(PMA)) shows distances within the PMA-treated group, and “type(between)” (e.g., seat(between)) those between paired samples with and without PMA treatment. **c** Principle coordinate analysis of MBTA samples using Bray-Curtis distances among filtered OTUs. Sample type and PMA treatment are both major drivers of overall community composition as expected

Based on this assessment, we applied PMA-seq to real-world microbial community samples from the Boston subway system as a representative (transit) built environment (Fig. 3). The relative abundances of several taxa changed significantly after PMA treatment (Additional file 6), but these PMA-reactive taxa varied among different sample types. As one example, the family Porphyromonadaceae was enriched consistently after PMA treatment in samples from seats and walls but remained stable in those from grips and touchscreens (Additional file 1: Figure S4).

Large compositional changes were observed in samples from seats and walls after PMA treatment, while grip and touchscreen communities exhibit less of a composition shift. This is arguably concordant with what we observed from the spiked samples and from previous studies [17], in which PMA treatment performed more effectively in less-complex samples with lower biomass (i.e., here, seats and walls). Likewise, Bray-Curtis dissimilarities differed more in walls and seats between PMA-free and PMA-treated samples (Fig. 3b). Independent of PMA treatment, sample type was again the main force driving taxonomic differences, explaining 37.8% of the variation (PERMANOVA FDR adjusted *p* = 0.002). Overall, and critically for future use of PMA-seq, our results indicate that microbial communities do not respond consistently to PMA treatment, with unique “PMA-reactive” taxa enriched after treatment in a context-dependent manner.

### “PMA-resilient” taxa are shared among studies while “PMA-responsive” taxa vary

A variety of factors are known to account for PMA responsiveness in complex communities [2]. To characterize common patterns of community structural changes upon PMA treatment, we compared our results with previous PMA-seq studies spanning three diverse environments: dust from a clean room environment, soil habitats, and stool [2, 5, 10]. We re-profiled each study’s raw data to ensure consistency (“Methods”) and identified the taxa with the greatest changes in relative abundance before and after PMA treatment in each sample type. Changes were calculated by dividing the relative abundance difference between PMA-free and PMA-treated samples by the initial relative abundance in PMA-free samples, allowing us to identify the taxa with the largest fold changes in response to PMA (i.e., PMA-responsive taxa).

Due to underlying differences in initial viability and community background, the change in the relative abundance of a microorganism was observed to be variable upon PMA treatment between sample types. Thus, PMA-responsive microbes were mostly unique in each independent source (Additional file 1: Figure S6). PMA treatment was able to remove several taxa (to undetectable) while also revealing others that were undetectable in the initial communities. On low-touch surfaces (cleanroom floors, computer screens, subway seats, and walls), the largest fold changes of relative abundances were mainly caused by the reduction of typically environmental taxa after PMA application, indicating that many non-viable microbes pertaining to those surfaces were successfully removed by PMA treatment. By contrast, the most responsive microorganisms on high-touch surfaces (computer mouse and subway grips), and in human stool and saliva samples, changed in the opposite direction after PMA treatment. These taxa, underrepresented or undetected in the PMA-free communities, were sometimes orders of magnitude more abundant after PMA treatment, which would indicate that they may be viable but rare organisms in these environments.

**Fig. 4:**
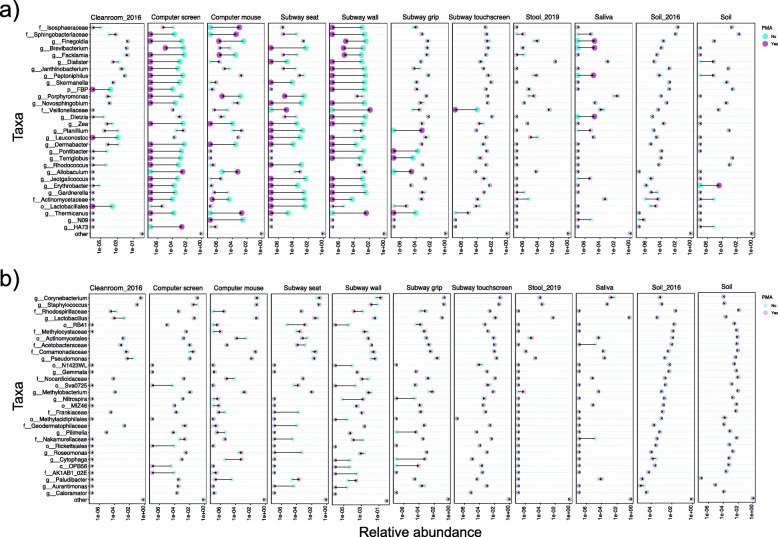
PMA treatment effects on different microbial community types in previous datasets compared with the present study. Relative abundances of 30 taxa with the **a** largest and **b** smallest fold changes across different sample types from previous PMA studies on clean room dusts (Cleanroom_2016) [5], soil (Soil_2016) [2], and stool (Stool_2019) [10] and our representative office and transit built environments. In each study, samples of the same type were combined, with the relative abundance of each taxon calculated by averaging its abundance in all samples of the same type. Each pair of PMA-free and PMA-treated samples is linked by arrows pointing from PMA-free to PMA-treated average relative abundance. Taxa are highlighted using larger, darker circles when changes in the relative abundance are larger than 100-fold after PMA-treatment

To better compare PMA treatment across different sample types, we ranked the most PMA-responsive taxa in each sample type and selected 30 taxa with the largest average relative abundance fold changes in at least two sample types (Fig. 4a). As expected, abundance changes of these taxa varied greatly in different samples. The abundance of the human commensal genus *Peptoniphilus* decreased from 10 to 2 to undetectable after PMA treatment on computer screens and subway walls, while remaining stable on clean room floors, computer mice, and subway grips. While in saliva and soil samples, it increased repeatedly from undetectable to a low fraction of ~ 10^−5^ after PMA treatment. Several other human commensals changed in similar directions across different sample types, such as the genera *Finegoldia* and *Gardnerella*, the Veillonellaceae family, and typically environmental microbes from the genus *Brevibacterium*. Biologically, these results suggest that functionally high-touch BE surfaces, intuitively, retain more viable human commensals compared with low-touch ones.

Using similar approaches, we also identified “PMA-resilient” taxa with the smallest average relative abundance fold changes within each sample type (Fig. 4b). In contrast to PMA reactivity, PMA resilience was more likely to be determined taxonomically, with less dependence on community background, biomass, or microbes’ abundance. Taxa of widely ranging abundances could be PMA-resilient (Fig. 4b, Additional file 1: Figure S7). Several highly abundant, human-derived genera, including *Corynebacterium*, *Staphylococcus*, and *Lactobacillus*, showed repeated stability upon PMA treatment in stool, saliva, soil, and on different BE surfaces. This would suggest that these abundant human commensals exist in viable forms in the BE; alternatively, they may simply be more resistant to PMA treatment. In summary, the above results suggest that PMA-based viability quantitation within communities may be both context-specific and taxon-specific, with “PMA-resilient” taxa often shared among similar ecosystem types while “PMA-responsive” taxa vary.

## Discussion

Although it is well-established that readouts of PMA-based viability can be highly context-dependent, this has been largely overlooked during its applications to complex microbial communities to date [2, 16]. This study performed a systematic evaluation of PMA-seq using synthetic, semi-synthetic, and environmental microbial communities, and for the first time explored its quantitative potential. PMA-seq was qualitatively appropriate for most settings, but not able to accurately quantify viable taxa in a generalizable way, even taking into account the inherent biases of amplicon sequencing and the variable biochemistry of PMA treatment. This was clear upon the comparison of newly profiled communities with re-analysis of existing PMA-seq data [6, 9, 20], which emphasized the degree to which microbial composition, biomass, and chemical environment (in addition to true viability) can all substantially influence results.

The efficacy of PMA treatment varied greatly in different environments, both qualitatively among samples and as quantified by known spike-in controls. This was true over and above the extent that amplicon sequencing often provides substantially skewed quantitative profiles in the first place, as noted above. We quantified this as an overall depletion efficiency of spiked dead microbes, i.e., the ratio between the amount of dead Enterobacteriaceae that was successfully removed after PMA treatment and the total amount of spiked Enterobacteriaceae. The resulting high efficacy in computer screens and computer mice somewhat explained their drastic compositional changes after PMA treatment. This can be considered a ceiling on the activity of PMA in any given environment since it considers only the degree to which PMA is affecting DNA known to be inactive. The high efficacy in soil samples (0.87) is an example of this and does not necessarily indicate an effective treatment, as the compositional dissimilarities between PMA-free and PMA-treated samples remained low (Fig. 2b). Moreover, the efficacy alone does not mean that different taxa were equally (un)affected. The low efficacy in saliva samples indicates the incomplete elimination of relic DNA, possibly attributed to a large amount of cell-free DNA from dead human cells in saliva samples, which are competitively bound by PMA molecules [21]. This observation echoes the results from previous studies, where incomplete relic DNA elimination after PMA treatment led to an overestimation of live bacteria and presented an “inflated” viable community [13, 17]. Meanwhile, unintended depletion of viable cells leads to false negatives of several microorganisms [16], especially those of low abundance.

Both the positive and negative aspects of these PMA behaviors in complex communities were reiterated in this study. Many apparently viable but low abundance microbes were overlooked in PMA-free communities, such as the *Allobaculum* genus and the Veillonellaceae family in the office and subway environments (Fig. 4a, Additional file 1: Figure S6). This is beneficial, inasmuch as failures to detect viable organisms in rigorously controlled environments such as the astronomical or medical industries can lead to serious or even lethal consequences. However, this advantage is offset by shortfalls, such as the ineffective removal of non-viable cells, which led to an inaccurate representation of viable communities. Some human-related genera such as *Finegoldia*, *Facklamia*, *Porphyromonas*, and *Dermabacter* were more than 100-fold less abundant after PMA treatment on computer mice, while barely changed on subway grips and touchscreens. Biologically, this would suggest that these genera are present in a more viable (or more membrane-intact) status on high-touch surfaces, where higher humidity and frequent human contact could both protect the membrane from rapid damage and/or frequently re-deposit new microbial cells with differing biochemical and adhesion properties. However, considering that the PMA treatment tends to be “overly effective” in computer mice (above calculation), it remains arguable whether these relative abundance decreases really represented a less-viable status or a toxic dose of PMA, especially when these clades were observed to be viable (or PMA-resilient) on similar high-touch surfaces. This fluctuating efficacy re-emphasizes that PMA behavior depends not only on the community but also on the membrane integrity status of different community members across environments, and ultimately no single quantitative method can be used universally even within similar sample sources, biomass ranges, or diversities. Instead, it must be calibrated to account for all these factors, with a fine line between “insufficient” and “overly effective.” This fragile balance makes it difficult to apply a protocol that consistently determines which microbes are viable across realistically complex community types, let alone quantifies them.

Many of the factors that influence PMA effectiveness have been well-explored in previous studies. In soil samples, for example, the mineralogy, pH, and ionic conditions all influence the longevity of relic DNA via adsorption of DNA molecules to soil matrices [2, 22], sequestering it from PMA intercalation [22, 23]. It is thus intuitive that higher PMA dye concentrations should be qualitatively appropriate in such samples, as compared with environments with a relatively clean, matrix-free background (e.g., swab samples). However, outside of extensive, environment-specific benchmarking (as per our experiments here), it is unclear how to estimate this effect a priori. This was reflected in our own soil data and its comparison with previous studies by the relatively small effects of PMA on any microbes (Figs. 2 and 4). Factors directly interfere with PMA’s chemical reactions also affect its efficacy [13, 24], such as turbidity, which influences optical density, thus affecting photoactivation [24, 25]. Light activation is also affected by the intensity of effective wavelength, and while we validated that the halogen lamp used here was adequate and consistent for PMA activation, it provides less optimal emission spectra compared with LEDs and UV lights now available [26–28]. The presence of organic content provides cation exchange sites that inactivate PMA [14, 25, 29]. These parameters all varied PMA performance in environmental samples, which are particle-rich and chemically heterogeneous. Even in the lab, membrane damage could be caused differentially among microbes by experimental practices such as centrifugal force and biomass loss during treatment, adding to measurement uncertainty. The biochemical heterogeneity of microbial community composition itself affects PMA efficacy, inasmuch as different cell wall structures, natural intake and efflux pump efficiencies, and DNA maintenance all influence its intracellular effects. This makes PMA somewhat more consistent in less diverse communities, as also observed in this study [16, 17, 24]. On the other hand, our study found these inconsistencies of PMA-seq to be somewhat consistent among similar environments, given the reproducibility of “PMA-resilient” microbes between datasets. Several human-derived PMA-resilient microbes were replicated in similar BE samples, suggesting that these abundant commensals are mostly viable on BE surfaces and are more resistant to PMA treatment. In light of this, PMA-seq may be used with greater confidence when comparing results between communities with similar actual viability, metabolic status, cell wall biochemistry, and cellular chemical environment [10, 30, 31].

A comprehensive quantification of PMA’s dependence on each of these parameters in microbial communities would require extensive effort, and there are limitations of the current study as a result. Our synthetically co-cultured and spiked “communities” use only a very small number of representative microbes, if anything leading us to underestimate the variability of PMA behavior between protocols and settings. Any such synthetic, high-biomass controls also open the door to contamination during sequencing, especially when accompanying low-biomass samples from the BE (e.g., Enterobacteriaceae and *Clostridia* in Fig. 2a and Additional file 1: Figure S2). While it may be impossible to design a universal PMA-seq protocol for perfect viability quantifications, the process optimized here is promising in terms of qualitative assessment: determining which microbes are generally more viable in diverse community settings. Other potential improvements might include sample pre-treatment by chemicals that alter community biochemical background [32, 33] or enzymes to selectively deplete host contamination [21, 34]. Perhaps most critically, viability assessment in microbial communities will benefit substantially from multi-omic integration, e.g., combining PMA-seq with functional indicators such as metatranscriptomic or metaproteomic profiles. These can circumvent some of PMA’s drawbacks, providing a complementary definition of viability, and directly observing activities such as virulence, pathogenicity, or antimicrobial resistance that are not captured by 16S sequencing.

Therefore, while PMA-seq alone may never fully quantify viability in microbial communities, it can provide a qualitative profile of which community members are generally viable, and remains to be coupled with additional molecular approaches to understand the mechanisms of persistence, metabolism, and potential health consequences in the BE, environmental, and human microbiomes [35, 36].

## Conclusions

This study evaluated the performance of PMA-seq as a viability screening method in synthetic and realistic complex communities, where it is as qualitatively appropriate as possible for most settings, detecting viable and non-viable microbes, but not able to accurately quantify viable taxa in a generalizable way. To our knowledge, this is the first systematic evaluation exploring the quantitative potential of PMA-seq. The inconsistent performance of PMA-seq reiterated that microbial composition, biomass, and chemical environment (in addition to true viability) can all substantially influence the results.

## Methods

### Preparation of synthetic microbial communities and *E. coli* spike-in culture

We constructed ten synthetic microbial communities (part 1) comprising viable or heat-killed *E. coli* strain ATCC 47076 and *S. sanguinis* strain ATCC 10556. The bacteria were sub-cultured on Brain Heart Infusion (BHI) agar and incubated overnight at 37 °C in room air (for *E. coli*) or 5% CO2 (for *S. sanguinis*). The bacteria were then inoculated into 5 ml fresh BHI broth and incubated at 37 °C while shaken at 250 rpm to reach early log-phase growth (OD_60_ = 0.1). The cultures were adjusted to 10^5^ CFU/ml by serial 10-fold dilution in BHI broth. For each of the strains, half of the cultures were killed by heat at 75 °C for 10 min (for *E. coli*) or 65 °C for 30 min (for *S. sanguinis)*. The heat-killed bacteria were then mixed with viable ones proportionally as shown in Fig. 1a.

We evaluated several killing conditions for the two strains (i.e., 65 °C, 70 °C, and 75 °C, incubated for 10 or 30 min) using plate counting and 16S rRNA qPCR. Among these, the optimal killing condition for *S. sanguinis* was 65 °C 30 min, where no bacterial growth was observed when streaking ~ 10^9^ cells on the plate, while the qPCR signal remained highest. This would suggest that the killing procedure itself causes minimal DNA degradation while inactivating the cells. Similarly, the killing condition for *E. coli* was optimized at 75 °C for 10-min incubation.

To calibrate an appropriate concentration of *E. coli* to be spiked into each community sample (part 2), we evaluated the bacterial load in community samples using qPCR of the 16S rRNA gene (primers and PCR details below), together with a series of 10-fold diluted *E. coli* cultures (10 to 10^8^ CFU/ml). The bacterial biomass (16S rRNA gene copies) on computer screens and computer mice was equivalent to 1 ml *E. coli* culture containing 10^2^–10^3^ cells, and soil and saliva equal to 10^6–^10^7^ cells (Additional file 1: Figure S2). Therefore, the low biomass samples (computer screens and computer mice) were spiked with ~ 500 *E. coli* cells (0.5 ml × 10^3^ CFU/ml), and high biomass samples (soil and saliva) were spiked with 10^6^ *E. coli* cells (1 ml × 10^6^ CFU/ml).

### Sample collection

Samples used in the spike-in experiment (part 2) were collected on July 8, 2019, from four separate computer screens, computer mice, soil environments, and human saliva. Each target was sampled in four replicates, one each to be spiked with *E. coli* culture and subjected to PMA treatment, an *E. coli* spike-in but PMA-free, a PMA-treated but no spike-in, and a plain control without any treatment. The computer screens and computer mice were sampled using four FLOQ swabs (Copan) in parallel. The swabs were pre-moisturized in autoclaved 0.85% saline. The entire surface of computer screens and computer mice were swabbed for 60 s (for one screen) or 30 s (for one mouse). The saliva samples were collected as described previously [37]. Briefly, the subjects were asked to let saliva collect in their mouth for one minute. Approximately 1.5 ml saliva was collected into a labeled 2-ml sterile tube. The soil samples were collected from four flowerpots around the Harvard T.H. Chan School of Public Health (655 Huntington Ave, Boston, MA). Four vials of the sample were obtained from each site, each containing 0.2 g of soil, for the different processing combination of PMA treatment and/or *E. coli* spike-in.

To assess BE microbial communities (part 3), we collected 16 samples from the Green Line on 19 August, 2019, including four samples each from the seats, walls, grips, and touchscreens of the ticket machines, largely as previously described [38]. The Massachusetts Bay Transportation Authority (MBTA) approved all aspects of our transit system sampling and gave permission to the Harvard T.H. Chan School of Public Health to conduct this study (Additional file 1: Figure S8). A sampling of the seats, grips, and walls was conducted in train cars as the train proceeded from the Longwood Station towards Park Street. Station samples were collected by swabbing the entire surface of touchscreens of ticket machines for one minute at the Park Street Station. Each surface was sampled by two pre-moisturized FLOQ swabs simultaneously, one PMA treated and one as an untreated control. For all the collections, swab heads were stored in 2-ml sterile tubes and placed on ice for no more than 1 h before being transported to the laboratory. Once at the lab, samples were transferred to a − 80 °C freezer for storage until processing.

### PMA treatment

Samples for PMA treatment (2 mM; Qiagen) were processed according to the manufacturer’s instructions. Briefly, samples were well mixed with PMA solution in 2-ml sterile tubes to make a final concentration of 10 μM (for synthetic community in part 1) or 50 μM (for all others) and placed in a dark drawer 10-min incubation under gentle rotation. PMA concentration for synthetic communities was adjusted to 10 μM based on the qPCR results (primers and PCR details below) on live and dead cultures (Additional file 1: Figure S1). After that, the tubes were placed horizontally atop a bed of ice and exposed to a 500-W halogen lamp at 20-cm distance for 10 min, inverting every 2 min to ensure homogenous exposure. Several different halogen lamps and LED light sources were evaluated in our pilot study, and the 500-W halogen lamp showed equivalent activation characteristics compared with commercial LED light sources when heat was controlled appropriately. The PMA-free samples were suspended in plain PBS buffer and incubated along with the PMA-treated samples. Pellets were harvested by centrifugation at 13,000*g* for 1 min followed by two washing steps using 500 μl of saline and were subsequently spinned down at the same speed.

### DNA extraction and real-time qPCR assay

Total microbial DNA was extracted using the DNeasy PowerLyzer PowerSoil Kit (Qiagen) following the manufacturer’s instructions, with concentrations quantified using a Qubit 2.0 fluorometer (Invitrogen, Carlsbad, CA). Real-time quantitative polymerase chain reaction (qPCR) was performed using universal primers, forward 5′-TACTACGGGAGGCAGCAG-3′ and reverse 5′-GGACTACCAGGGTATCTAATCCTGTT-3′, to amplify a 466-bp region in the 16S rRNA gene [39] in the synthetic community (part 1) and spike-in experiment (part 2). Each 20 μl reaction mixture consisted of 10 μl 2X KAPA SYBR FAST qPCR Master Mix (KAPA Biosystems), 0.4 μl (a final concentration of 10 μM) forward and reverse primers, 8.2 μl PCR grade water and 1 μl of DNA template (for bacterial cultures, soil and saliva samples), or 4.2 μl PCR grade water and 5 μl of DNA template (for the computer screen and computer mouse surfaces). The thermocycling program was as follows: (1) initial denaturation for 3 min at 95 °C, (2) 40 cycles of 3 s at 95 °C and 20 s at 60 °C, followed by (3) a melting curve in the range of 60 °C to 95 °C. Standard curve was generated in each batch of run using serial-dilutions of pSPIKE-P(Addgene Plasmid #101172), a plasmid of known size and with 16S rRNA V4 region insertion.

### 16S rRNA gene amplicon sequencing

The 16S rRNA gene sequencing protocol was adapted from the Earth Microbiome Project [40] and the Human Microbiome Project [41]. In brief, genomic DNA was subjected to 16S amplification using primers incorporating the Illumina adapters and a sample barcode, allowing directional sequencing over 16S gene variable region V4. Each 25 μl PCR reaction contained 10 μl of 2X HotMasterMix with the HotMaster Taq DNA Polymerase, and 5 μl of primer mix (2 μM of Forward primer 515F and 2 μM barcoded Reverse primer 806R). The thermocycling conditions comprising an initial denaturation of 94 °C for 3 min, followed by 30 cycles of denaturation at 94 °C for 45 s, annealing at 50 °C for 60 s and extension at 72 °C for 90 s, and a final extension at 72 °C for 10 min. Sequencing was performed on the Illumina Miseq platform according to the manufacturer’s specifications with the addition of 15% PhiX and yielded paired-end reads of 150 bp in length in each direction.

### Sequencing data analysis

We obtained a median of 48,437 (average 91,861) reads per sample from the sequencing. Taxonomic profiles were generated with the bioBakery 16S workflow (version 0.12.1) built with AnADAMA2 [42], which incorporates EA-Utils [43] and the UPARSE pipeline (version 8.1) [44]. In brief, paired-end reads were first demultiplexed to remove barcodes using the fastq-multx command in EA-Utils (version 1.1.2). After that, reads were merged with the fastq_mergepairs command, and filtered to remove low-quality sequences with UPARSE threshold of *E*_max_ = 1, at which the most probable number of base errors per read is zero after filtration, and a truncation quality threshold of 15. Further reads were trimmed to a fixed length of 200 bp. Singletons and chimera reads were removed while simultaneously grouping into operational taxonomic units (OTUs) at 97% similarity using USEARCH (version 7.0.1090) [45]. Then, phylogenetic trees are constructed after centroid sequence alignment using Clustal Omega [46]. Finally, the representative sequences for each cluster were mapped against the Greengenes database (version 13.8) [47] to build into an OTU feature table.

### PMA efficacy calculation

In the present study, we defined the PMA efficacy by calculating the amount of dead reference (typically *E. coli*) removed by PMA divided by total spike-in:

PMA efficacy = 1-(D-B)/(C-A)

A: Abundance of Enterobacteriaceae × 16S rRNA copies in plain sample

B: Abundance of Enterobacteriaceae × 16S rRNA copies in PMA-treated sample

C: Abundance of Enterobacteriaceae × 16S rRNA copies in sample with *E. coli* spike-in

D: Abundance of Enterobacteriaceae × 16S rRNA copies in sample with *E. coli* spike-in and PMA-treatment

As the spike-in culture was composed of 1:1 live:dead *E. coli* cells, the efficacy should theoretically equal to 0.5—half of the community cells are dead and removed by PMA, with no live cells affected. An efficacy > 0.5 indicates that some viable cells are also removed by the treatment; while an efficacy < 0.5 means the incomplete removal of relic DNA molecules. The calculation used the relative abundance of the entire Enterobacteriaceae family, into which this UPARSE/Greengenes workflow places *E. coli* non-specifically (though the Enterobacteriaceae family is overwhelming dominated by a single OTU highly likely to be *E. coli*, Additional file 1: Figure S9). The PMA efficacy in a sample type was calculated by averaging the Enterobacteriaceae abundance in all the individual samples of that type, times the averaged log_10_ 16S rRNA gene copies obtained from 16S qPCR assays.

### Statistical analyses

An in-house R-script employing the libraries stringr, dplyr, matrixStats, vegan, ape, ggplot2 [48], phytools [49], scales, phyloseq [50] and GUniFrac [51] was used to compare the outputs from bioBakery workflows (Additional file 2). First, OTUs were condensed to the genus level if possible; when a genus was not assigned, a sum abundance was calculated at each OTU’s terminal taxonomic level. The resulting clades are referred to generally as taxa. Taxa were then passed through a filter of > 0.01% relative abundance in at least 10% of all samples to fix the zero-inflation issue and to exclude extreme outliners or sequencing errors. Principal coordinate analysis (PCoA) was performed using Bray-Curtis dissimilarity based on these relative abundances. Univariate tests were performed using PERANOVA for differentially abundant taxa with respect to sample types, PMA treatment and spike-in effect (for part 2). Multivariate tests for taxa associated with metadata (Additional file 3) were performed using MaAsLin 2 [52]. For this analysis, we included three types of metadata as covariates: sample type, PMA treatment, and spike-in (for part 2 samples).

## Supplementary Information

**Additional file 1: Figure S1.** Determining PMA working concentration in *E. coli* and *S. sanguinis* cultures. **Figure S2.** Determining microbial biomass of community samples relative to different concentrations of *E. coli* cultures. **Figure S3.** Taxonomic composition of control samples (n=11). **Figure S4.** Relative abundance of Porphyromonadaceae family with and without PMA treatment in subway samples. **Figure S5.** Summary of the 606 samples from four datasets used for comparative analysis. **Figure S6.** Taxa most affected by PMA treatment samples in previous studies compared to the office built environment, saliva, and Boston subway. **Figure S7.** Taxa least affected by PMA treatment in previous studies compared to the office built environment, saliva, and Boston subway. **Figure S8.** Approval from the MBTA. **Figure S9.** Evidence that the spike-in portion of *E. coli* is likely very similar in sequence to those strains used during the synthetic experiment.

**Additional file 2.** bioBakery workflows.

**Additional file 3.** Multivariate tests for taxa associated with metadata.

**Additional file 4.** PERMANOVA FDR adjusted.

**Additional file 5.** Metadata.

**Additional file 6.** Relative abundances of several taxa changed significantly after PMA treatment.

**Additional file 7:.** All sample taxonomy.

**Additional file 8:.** All sample meta.

## Data Availability

The datasets supporting the conclusions of this article are included in the additional files (Additional files 1, 2, 3, 4, 5, 6, 7 and 8) of this article. The raw sequencing data are available in the NCBI SRA repository, under BioProject number: PRJNA648844.
