# Supplementary material for: Whole microbial community viability is not quantitatively reflected by propidium monoazide sequencing approach
Source: Microbiome. 2021 Jan 21;9:17. doi: 10.1186/s40168-020-00961-3 (PMC7819323; doi:10.1186/s40168-020-00961-3)
Supplement: Supplementary file 2 — Additional file 1: Figure S1. Determining PMA working concentration in E. coli and S. sanguinis cultures. Figure S2. Determining microbial biomass of community samples relative to different concentrations of E. coli cultures. Figure S3. Taxonomic composition of control samples (n=11). Figure S4. Relative abundance of Porphyromonadaceae family with and without PMA treatment in subway samples. Figure S5. Summary of the 606 samples from four datasets used for comparative analysis. Figure S6. Taxa most affected by PMA treatment samples in previous studies compared to the office built environment, saliva, and Boston subway. Figure S7. Taxa least affected by PMA treatment in previous studies compared to the office built environment, saliva, and Boston subway. Figure S8. Approval from the MBTA. Figure S9. Evidence that the spike-in portion of E. coli is likely very similar in sequence to those strains used during the synthetic experiment. [file 40168_2020_961_MOESM2_ESM.pdf]

Whole microbial community viability is not quantitatively reflected by propidium monoazide sequencing approach

Supplementary figures:

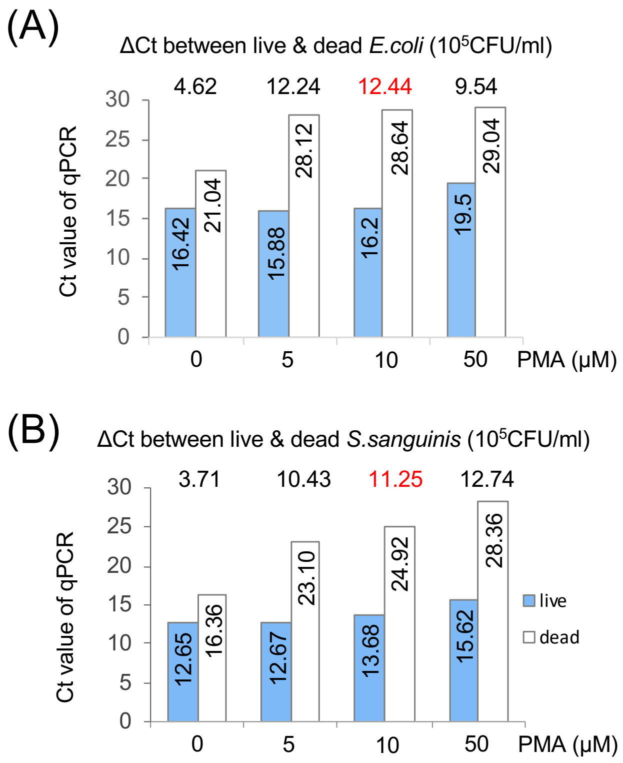

Figure S1: Determining PMA working concentration in *E. coli* and *S. sanguinis* cultures. qPCR was performed targeting 16S rRNA gene in 10^5 CFU/ml viable or heat-killed *E. coli* and *S. sanguinis* cultures with different concentrations of PMA treatment. An ideal working concentration is where the biggest Ct value differences are observed between live/dead cells, meanwhile the Ct value of live cells does not increase apparently (acceptable impact on viable cells). 10μM was selected here for the simple culture experiment.

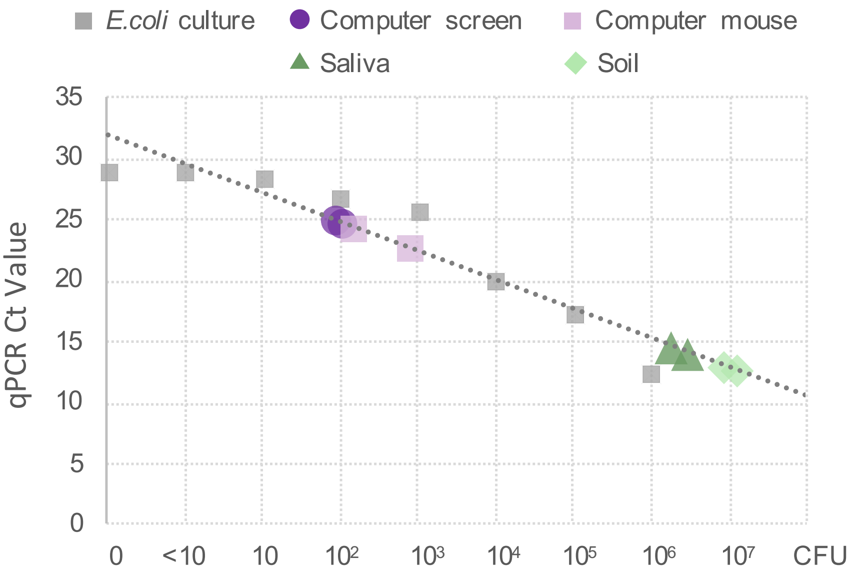

Figure S2: Determining microbial biomass of community samples relative to different concentrations of *E. coli* cultures. qPCR was performed targeting 16S rRNA gene V4 region in two computer screen surface samples, two computer mice, two human saliva and two soil samples, together with eight 10-fold serial dilutions of *E. coli* cultures. Bacterial mass in computer screens and computer mice samples are close to 10^2 and 10^3 CFU/ml *E.coli* culture, and saliva and soil equal to 10^6 and 10^7 CFU/ml.

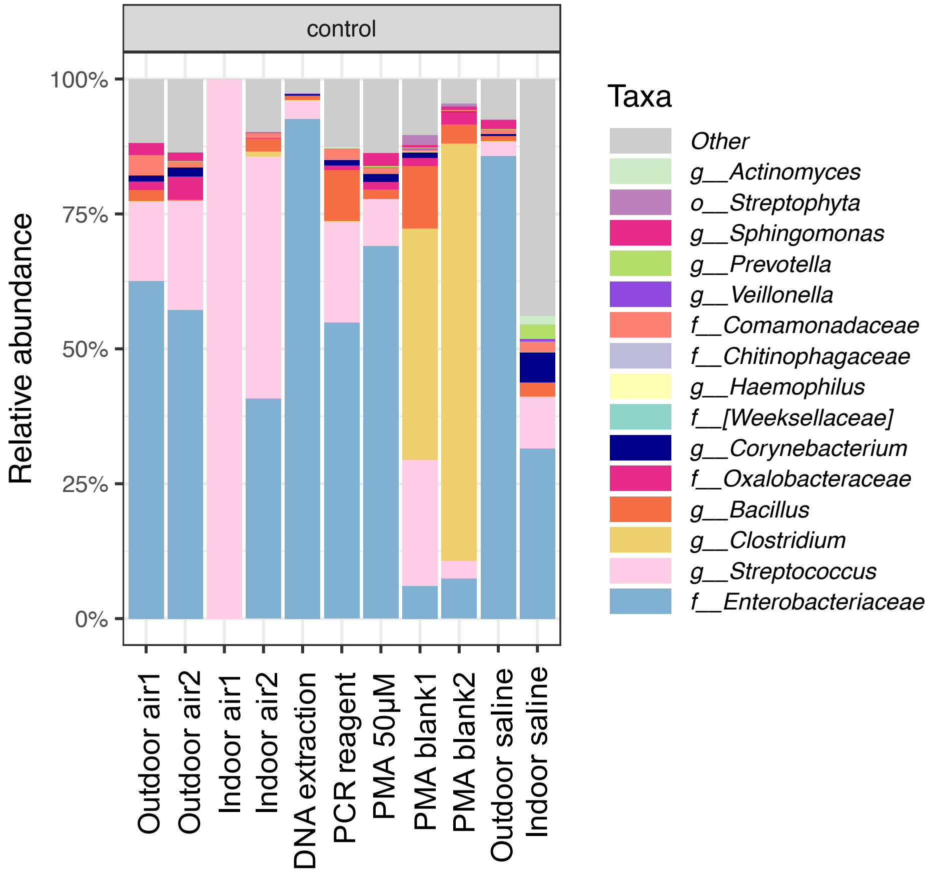

Figure S3: Taxonomic composition of control samples (n=11). Relative abundance of eleven control samples is presented here, prepared from two outdoor air, two indoor air, one blank DNA extraction, one blank library-preparation PCR reagent, one PMA 50 µM solution, one saline used for swab moistening in indoor environment and and one in the outdoor environment.

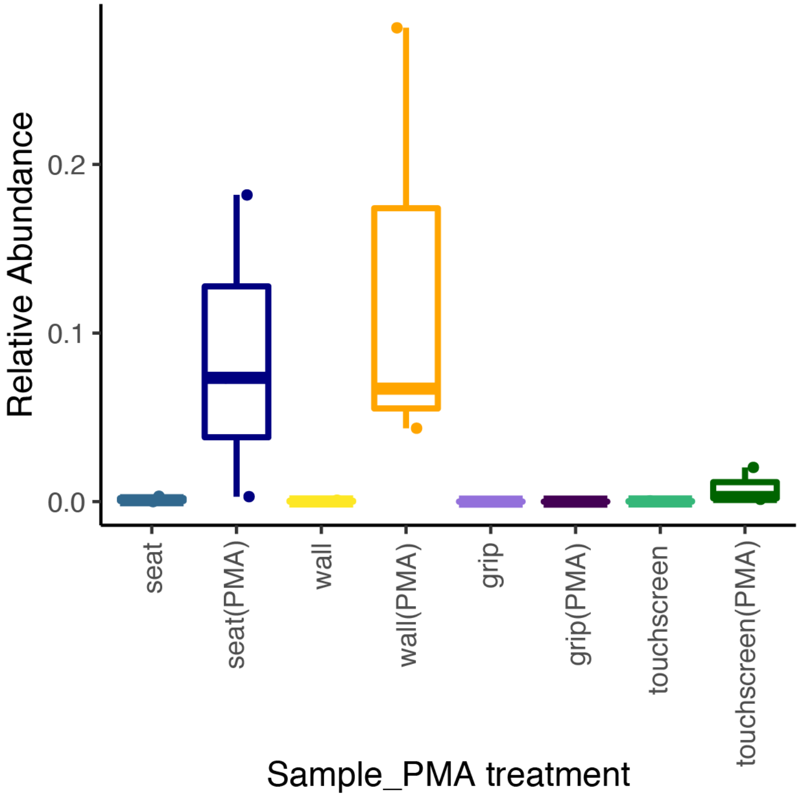

Figure S4: Relative abundance of Porphyromonadaceae family with and without PMA treatment in subway samples. Each column represents the relative abundance of Porphyromonadaceae family in samples with (labelled as sample(PMA) or without PMA treatment.

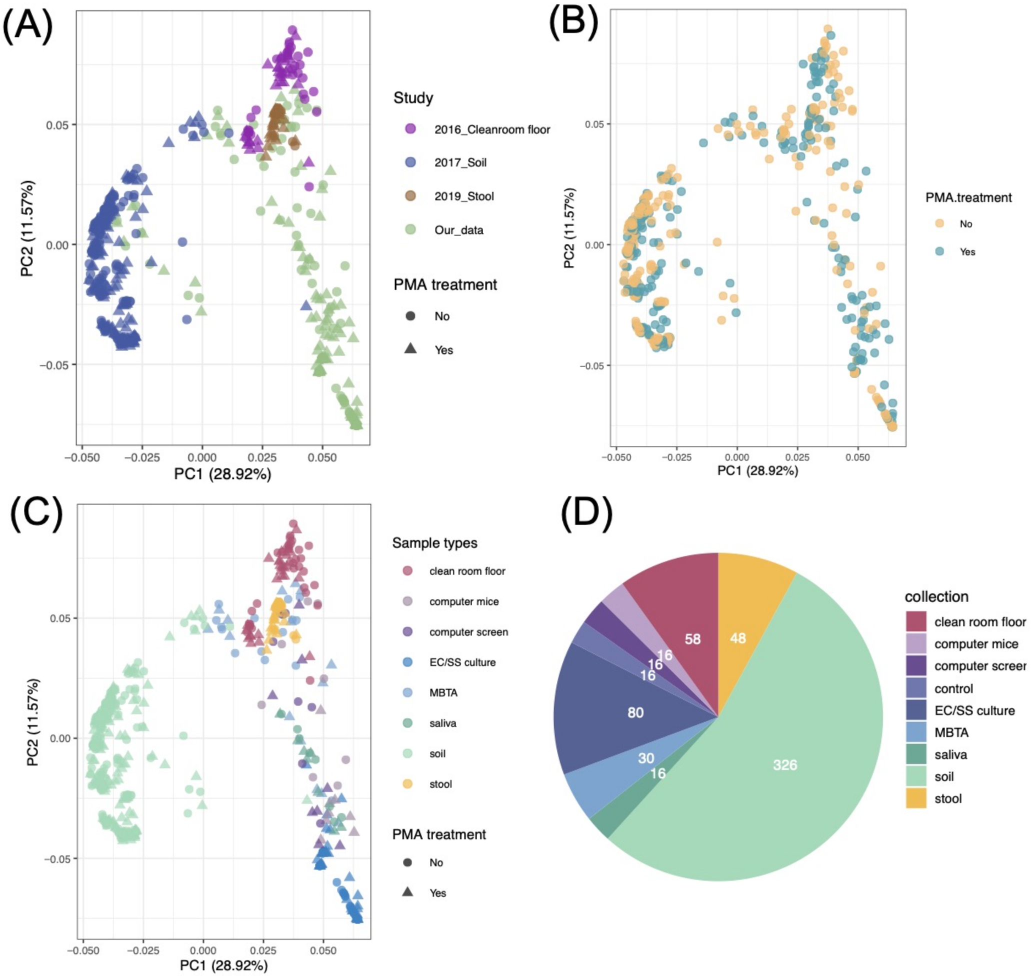

Figure S5: Summary of the 606 samples from four datasets used for comparative analysis. A total of 606 samples from the 2016 clean room dusts study (n=58), the 2016 soil study (n=310), the 2019 faecal transplantation study (n=48), and from our current study (n=190). Principal coordinate analyses were carried on all the samples using Bray-Curtis distances among filtered OTUs, colored by datasets (A), PMA treatment (B) or sample types (C). (D) summarized the sample compositions across the four studies.

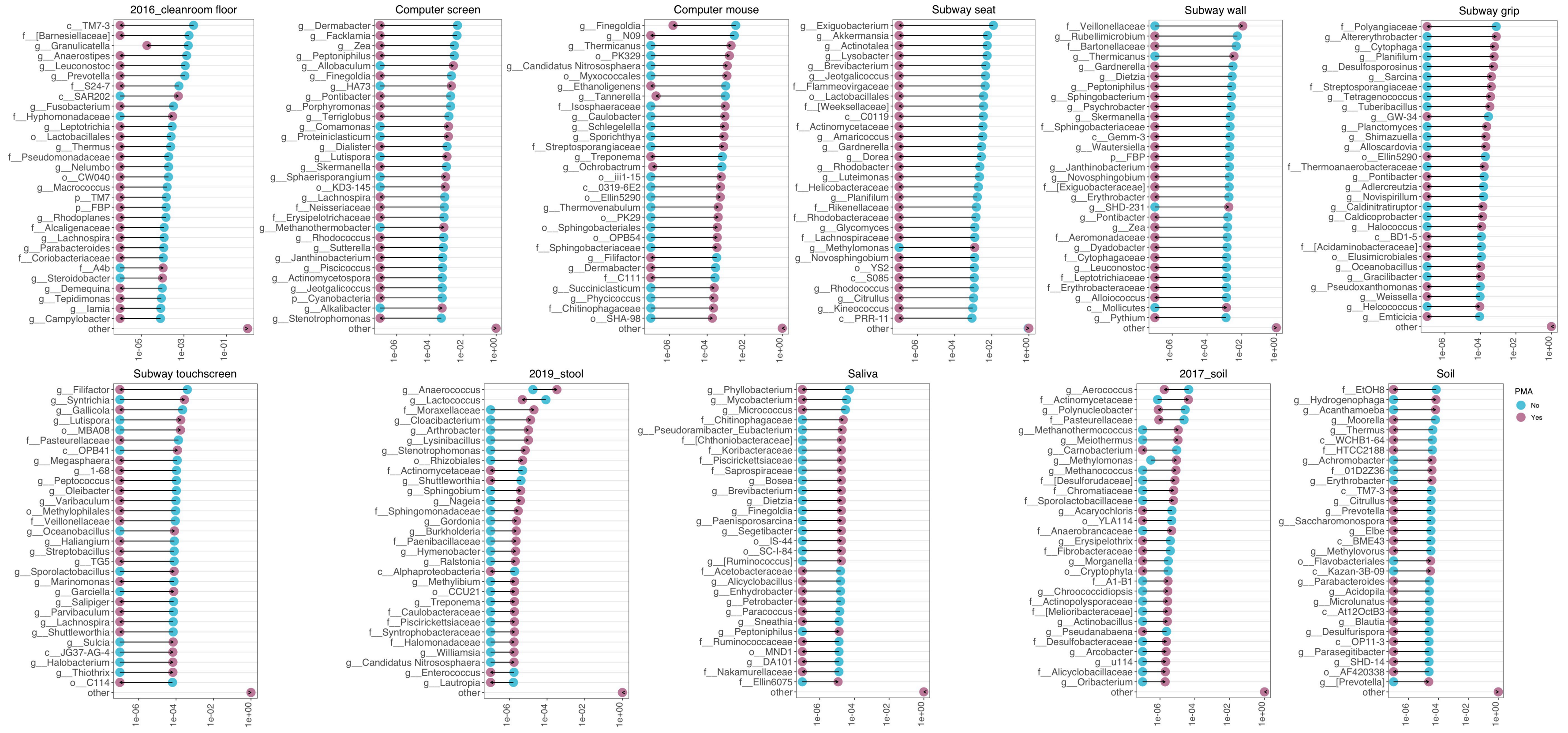

Figure S6: Taxa most affected by PMA treatment samples in previous studies compared to the office built environment, saliva, and Boston subway. Relative abundances of 30 taxa with the largest abundance fold changes in each sample type from three previous studies and our representative and transit built environments are shown. In each sample type, relative abundance of a taxon was calculated by averaging its abundance in all the samples without PMA treatment (PMA-free abundance) or after PMA treatment (PMA-positive abundance). X axis showed the relative abundance. Dots denote the relative abundance of each taxon in PMA-free (blue) and PMA-treated (purple) samples. The average abundances in PMA-free and PMA-treated samples were linked by arrows, pointing from PMA-free samples to the PMA-treated ones.

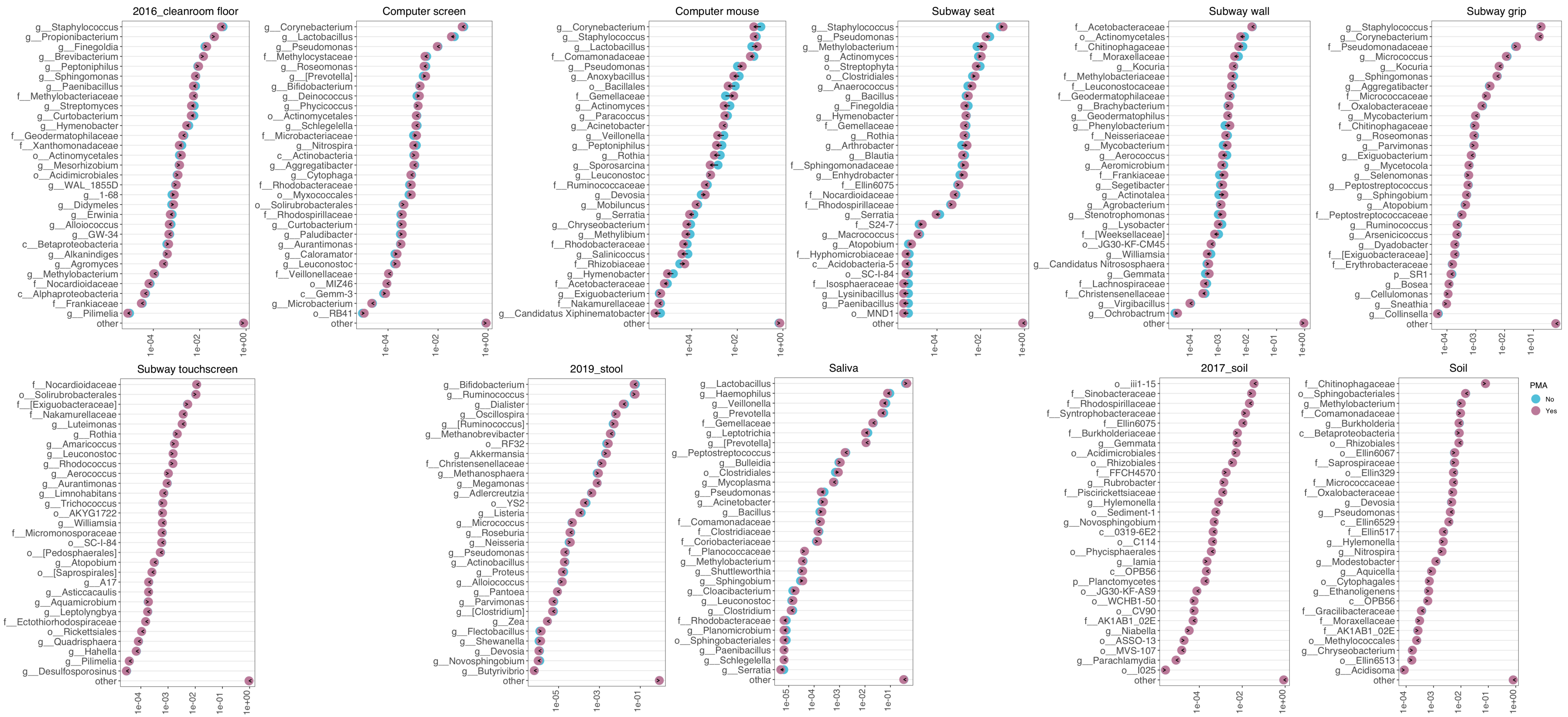

Figure S7: Taxa least affected by PMA treatment in previous studies compared to the office built environment, saliva, and Boston subway. Relative abundances of 30 taxa with the smallest abundance fold changes in each sample type from three previous studies and our representative and transit built environments are shown. In each sample type, relative abundance of a taxon was calculated by averaging its abundance in all the samples without PMA treatment (PMA-free abundance) or after PMA treatment (PMA-positive abundance). X axis showed the relative abundance. Dots denote the relative abundance of each taxon in PMA-free (blue) and PMA-treated (purple) samples. The average abundances in PMA-free and PMA-treated samples were linked by arrows, pointing from PMA-free samples to the PMA-treated ones.

Charles D. Baker, Governor
Karyn E. Polito, Lieutenant Governor
Stephanie Pollack, MassDOT Secretary & CEO
Brian Shortsleeve, Chief Administrator and Acting General Manager

massDOT
Massachusetts Department of Transportation

MBTA Onsite Activities Permit

The Massachusetts Bay Transportation Authority ("Authority") hereby grants permission to

Harvard Microbiome Sampling Team ("Permit Holder"),

with a usual place of business/residence at Harvard T.H. Chan School of Public Health

to enter upon its right of way, premises or structures at the following Authority locations for the purposes of public health research ("Permitted Activity") on the following dates and times:

STATION	DATE	TIME
Roxbury Crossing and Longwood Medical	8/19 - 8/26	As needed

NOTE:

The sampling date may be changed due to unexpected reason, changed as needed.

The Permitted Activity is ☐ Commercial ■ Non-Commercial

This permit is subject to and incorporates by reference herein the terms and conditions of the General Release entered into between the Authority and Permit Holder concurrent with the issuance of this Permit. By accepting the privileges of this Permit, the Holder accepts and agrees to abide by those Terms, and to comply with the following.

This permit is not assignable or transferable and may be cancelled at any time at the discretion of the Authority. The Permit Holder must keep this permit with them at all times while engaging in the Permitted Activity. .Failure to produce this permit for inspection at the request of Authority personnel may subject the Permit Holder to revocation of this Permit and ejection from Authority property without recourse.

Permission to engage in the Permitted Activity is granted on the express condition that the Permit Holder does not interfere with the Authority's operations. At all times, the Permit Holder shall abide by the rules, regulations, orders and/or directions of Authority officials. Permit Holders are instructed to find a Customer Service Agent or Supervisor for placement regarding commercial activity.

All Permit Holders shall conduct themselves in a friendly, non-aggressive manner so as not to inconvenience or annoy customers, delay trains, or compromise safety on or near yellow safety lines or in rail pits.

All questions regarding this Permit shall be directed to MBTA Marketing Communications, 10 Park Plaza, Room 7500 Boston, MA 02116. Phone 617-222- 5568; Email: marketingmanager@mbta.com

Approved by:

Issue Date 7/19/2019

MASSACHUSETTS BAY TRANSPORTATION AUTHORITY
TEN PARK PLAZA, SUITE 3910, BOSTON, MA 02116
WWW.MBTA.COM

Figure S8: Approval from the MBTA. We received the letter of approval from MBTA, by way of the General Manager's Office, to carry out the study and confirmed the detailed sampling plans with the MBTA prior to any public work. Their assistance and input were invaluable both for study design and for safe execution of sample collection, and the letter includes the initial information from Evan Rowe approving the work.

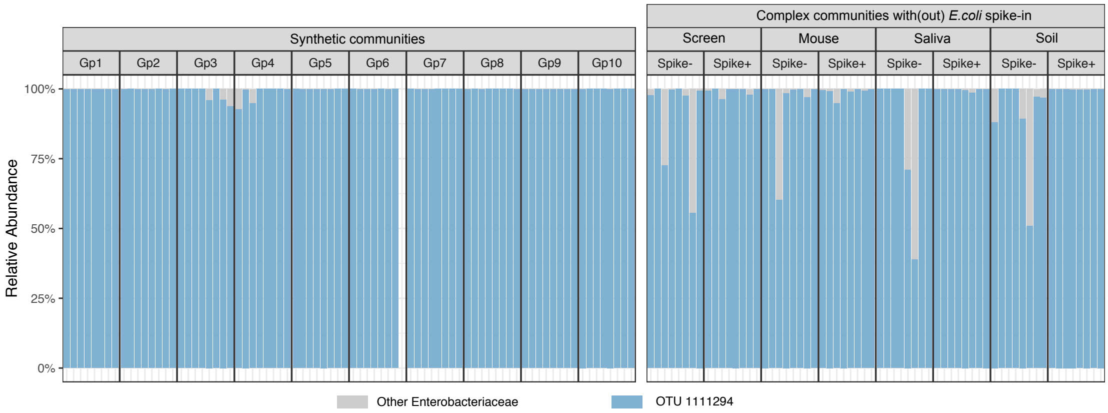

Figure S9: Evidence that the spike-in portion of E. coli is likely very similar in sequence to those strains used during the synthetic experiment. Relative abundance of OTU 1111294 (*E. coli*) in total reads assigned to Enterobacteriaceae family across different sample types.
